# LOR Regulated by METTL3 Alleviates Lipopolysaccharides-Induced Periodontitis Injury

**DOI:** 10.4014/jmb.2505.05016

**Published:** 2025-08-18

**Authors:** Qin Su, Jiao Chen

**Affiliations:** Department of Stomatology, Wuhan Fourth Hospital, Wuhan 430033, Hubei, P.R. China

**Keywords:** Periodontitis, LPS, METTL3, LOR, m^6^A modification

## Abstract

Periodontitis is a chronic inflammatory disease-causing tissue destruction and systemic effects. Despite significant advancements, the molecular mechanisms driving tissue degeneration remain incompletely understood. Emerging evidence suggests that RNA modifications, particularly N6-methyladenosine (m^6^A) methylation, critically regulate inflammatory responses. This study investigates the role of METTL3-mediated m^6^A modification of loricrin (LOR) in lipopolysaccharide (LPS)-induced periodontal injury. Bioinformatics analyses identified the key downregulated gene in periodontitis. To establish an *in vitro* periodontitis model, human periodontal ligament fibroblast (HPLF) cells were treated with LPS. LOR and METTL3 levels in clinical samples and HPLF cells were measured by qRT-PCR. Inflammatory cytokines, cell proliferation, and apoptosis were examined using ELISA, CCK8, EdU, and flow cytometry assays, respectively. The interaction between LOR and METTL3 was evaluated through Pearson correlation, MeRIP assay, qRT-PCR, immunoblotting, and mRNA stability assays. LOR was identified as a key downregulated gene in periodontitis, as validated in both clinical tissues and a periodontitis cell model. Functional assays showed that LPS-treatment promoted inflammatory cytokine production, inhibited cell proliferation, and increased apoptosis, whereas upregulating LOR in these cells reversed these effects. Furthermore, METTL3 expression was reduced in periodontitis clinical tissues and positively correlated with LOR expression. METTL3 overexpression enhanced LOR mRNA stability via m^6^A methylation. Moreover, silencing METTL3 partially negated the protective effects of LOR overexpression in LPS-induced periodontitis cell model. These findings reveal that METTL3-mediated m^6^A modification of LOR mitigates periodontal injury, suggesting that the METTL3-LOR axis may represent a potential avenue for future therapeutic exploration to maintain periodontal homeostasis.

## Introduction

Periodontitis is a chronic inflammatory condition marked by the deterioration of periodontal tissues that in due course results in tooth loss [1]. This disease is multifactorial, associated with genetic predispositions, environmental factors such as smoking and diet, and microbial factors, primarily plaque biofilms [2-4]. Lipopolysaccharide (LPS) is an important part of the outer membrane of Gram-negative bacteria and serves a central role in the pathogenesis of periodontitis [5]. LPS has been established to initiate and perpetuate tissue destruction by stimulating an inflammatory response in periodontal ligament fibroblasts and other resident cells [6, 7]. Although considerable progress has been made in identifying clinical risk factors and therapeutic interventions [8], a comprehensive understanding of periodontitis pathobiology remains elusive. Therefore, elucidating these mechanisms is critical for the development of innovative approaches for early diagnosis, targeted therapy, and improved patient outcomes.

Recent research highlights the critical role of epigenetic regulation in various diseases, including periodontitis [9]. Epigenetics refers to the regulation of gene expression and cell differentiation through modifications that do not alter the DNA sequence, affecting gene activity and cellular phenotype [10]. The main epigenetic mechanisms encompass chromatin remodeling, non-coding RNAs, histone modifications, DNA methylation, and RNA modifications [11]. Among these, N6-methyladenosine (m^6^A) is the most widespread RNA modification in eukaryotic cells, occurring at the N6 position of adenosine, and plays a pivotal role in regulating biological functions and contributing to various diseases [12]. It is a dynamic and reversible process primarily controlled by methyltransferases, demethylases, and proteins that recognize methylation [13]. Methyltransferase-like 3 (METTL3) is a core component of the m^6^A methyltransferase complex, primarily responsible for catalyzing the methylation of adenosine residues on RNA molecules [14]. Emerging evidence suggests that METTL3 may participate in periodontitis pathogenesis by modulating the inflammatory microenvironment. For instance, METTL3 played a crucial role in promoting ribosome biogenesis and oxidative phosphorylation in osteoblasts by activating the Wnt/β-catenin/c-Myc signaling pathway, which helped mitigate periodontitis inflammation and additional alveolar bone loss [15]. Furthermore, METTL3 has been shown to enhance osteogenic differentiation in periodontal mesenchymal stem cells by modulating lncRNA CUTALP, which inhibited miR-30b-3p and activated Runx2, contributing to better osteogenesis and potentially alleviating periodontitis progression [16]. Another study reported that METTL3 promoted osteogenic differentiation of human periodontal ligament stem cells in an inflammatory niche by enhancing miR-141-3p levels through m^6^A modification, which suppressed ZEB1 and helped counteract periodontitis-induced osteogenic impairment [17]. Despite significant advances in understanding the pathogenesis of periodontitis, the role of RNA modifications, particularly METTL3-mediated m^6^A methylation, remains underexplored.

In this study, we examined the role and mechanisms of METTL3 in periodontitis. We specifically explored the interactions between METTL3 and Loricrin (LOR), a gene identified through bioinformatics analysis as a downregulated gene in periodontitis. It is a vital structural protein in the cornified cell envelope, is essential for maintaining epithelial integrity and function [18]. However, little is known about how LOR expression is regulated at the post-transcriptional level during periodontal disease, especially through epigenetic mechanisms like m^6^A methylation. By linking m^6^A methylation to epithelial barrier dysfunction and inflammation, our work not only expands the understanding of m^6^A biology in periodontitis but also indicates possible future strategies to strengthen the epithelial barrier and prevent disease progression.

## Materials and Methods

### Bioinformatics Analyses

Downregulated differentially expressed genes (DEGs) from the GSE10334 dataset were identified using criteria of adj. *P* < 0.01 and logFC < -1. This dataset, consisting of 183 periodontitis and 64 healthy samples, was filtered to select the downregulated genes in periodontitis. The top 30 downregulated genes were then analyzed using STRING for protein-protein interaction (PPI) analysis, and the gene with the highest connectivity in the PPI network was selected for further investigation.

### Clinical Samples

Gingival tissue samples were obtained from patients diagnosed with periodontitis and healthy controls undergoing tooth extraction for orthodontic purposes at Wuhan Fourth Hospital. Informed consent was obtained from all participants, and the study was approved by the ethics committee of Wuhan Fourth Hospital (approval number: KY2024-015-04). Clinical characteristics, including age, sex, dietary favor, gingival index, plaque index, probing pocket depth, and clinical attachment level, were noted in Table S1.

### Cell Culture and Transfection

The human periodontal ligament fibroblast (HPLF) cells (CP-H136, China) were cultured in human periodontal fibroblast complete culture medium (CM-H136, Procell) at 37°C in a humidified atmosphere containing 5% CO_2_. To simulate periodontitis injury without causing excessive cytotoxicity, cells at approximately 80% confluence were treated with LPS (1 μg/ml, Sangon Biotech, China) for 24 h, as described in a previous study [19].

The overexpression vectors of LOR and METTL3 (OE-LOR and OE-METTL3) were constructed by RiboBio (China) using the pcDNA3.1 vector, with an empty vector serving as a negative control (NC). The siRNA targeting METTL3 (si-METTL3) and the corresponding NC (si-NC) were also obtained from RiboBio. For the transfection of siRNAs and vectors into cell lines, Lipofectamine 3000 (Thermo Fisher Scientific, USA) was used following the manufacturer's instruction.

### Quantitative Real Time PCR (qRT-PCR)

Total RNA was extracted from tissue samples and cultured cells using TRIzol reagent (Invitrogen, USA). Complementary DNA (cDNA) synthesis was performed using a PrimeScript RT Reagent Kit (Takara, Japan). Thereafter qRT-PCR was carried out using the SYBR Green PCR kit (Qiagen, Germany). The data were quantitatively analyzed utilizing the 2^−ΔΔCt^ method with GAPDH serving as an internal control. Primer sequences used for LOR, METTL3, and GAPDH are listed in Table S2.

### ELISA Assays

Concentrations of pro-inflammatory cytokines (interleukin-1β (IL-1β), interleukin-6 (IL-6), and tumor necrosis factor-α (TNF-α)) were measured in cell culture supernatants utilizing commercial ELISA kits (Beyotime, China) following the manufacturers' guideline.

### CCK8 Assay

The LPS treated and transfected HPLF cells were inoculated onto 96-well plates at a density of 1 × 10³ cells/well. After 48 h of transfections, 10 μl of CCK-8 reagent (Beyotime) was added to each well for a 2-h incubation at 37°C. Lastly, the absorbance values were recorded using a microplate reader (Hiwell Diatek, China) at 450 nm.

### EdU Incorporation Assay

Cell proliferation was evaluated using an EdU Cell Proliferation Kit (RiboBio). Briefly, cells were incubated with EdU reagent for 2 h, fixed, permeabilized, and stained. EdU-positive cells were visualized using a fluorescence microscope, and the percentage of proliferating cells was calculated.

### Flow Cytometry for Apoptosis Detection

Cell apoptosis was evaluated via Annexin V-FITC/propidium iodide (PI) staining (Solarbio, China). After treatments, cells were collected, stained with Annexin V-FITC and PI, and analyzed by flow cytometry (BD Biosciences, USA).

### Western Blotting

Proteins were extracted using RIPA lysis buffer with protease inhibitors, and protein amount was quantified using a BCA Protein Assay Kit (Thermo Fisher Scientific). The proteins were separated by SDS-PAGE and transferred onto PVDF membranes. The membranes incubated with 5% non-fat milk for blocking and then exposed to primary antibodies against LOR (A21039, ABclonal, China), METTL3 (A19079, ABclonal), and GAPDH (A19056, ABclonal) overnight at 4°C. On the following day, the membranes were treated with a goat-derived HRP-conjugated secondary antibody (AS014, ABclonal) for 1 h and the bands were seen by employing ECL reagents (Beyotime).

### Methylated RNA Immunoprecipitation (MeRIP) Assay

The MeRIP assay was performed by Magna MeRIP m^6^A Kit (Millipore, USA). Briefly, total RNA was extracted from cells stably transfected with either OE-METTL3 or OE-NC vectors. The RNAs were then chemically fragmented using fragmentation reagents and immunoprecipitated using protein A/G Sepharose beads conjugated with either anti-m^6^A antibody or IgG antibody (NC). Following that, enriched mRNAs were purified and analyzed by qRT-PCR to detect LOR methylation levels.

### mRNA Stability Assay

To assess mRNA stability, transfected HPLF cells were exposed to actinomycin D (5 μg/ml, Sigma, USA) to inhibit transcription. Total RNA was harvested at 0, 4, and 8 h post-treatment, and relative LOR mRNA levels were quantified by qRT-PCR.

### Statistical Analysis

All experiments were performed in triplicate. Data are presented as the mean ± standard deviation (SD). Statistical analyses were conducted using GraphPad Prism 8.0 (GraphPad Software, USA). Differences between groups were evaluated using Student's *t*-test (two groups) or one-way ANOVA (multiple groups). Correlation analysis between LOR and METTL3 was performed by Pearson correlation coefficients. *P*-values < 0.05 were considered statistically significant.

## Results

### LOR Was Identified as a Key Downregulated Gene in Periodontitis

**Fig. 1:**
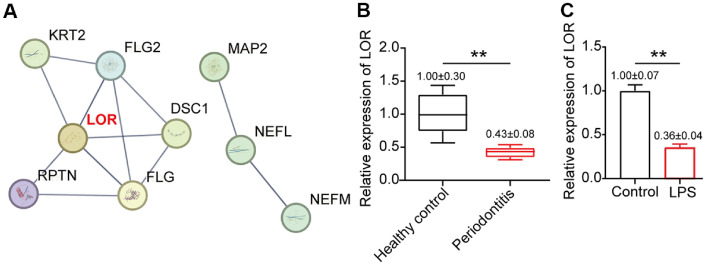
LOR was identified as a key downregulated gene in periodontitis. (**A**) The top 30 downregulated genes from GSE10334 were analyzed using the STRING database to construct a protein-protein interaction (PPI) network for identifying key regulatory candidates. (**B**) The mRNA expression of LOR was evaluated by qRT-PCR in gingival tissues from periodontitis patients and healthy controls. (**C**) The LOR expression was analyzed in LPS-treated HPLF cells (an *in vitro* periodontitis model) by qRT-PCR. All data (sample size = 3), obtained from three biological replicates, are presented as mean ± SD. **P < 0.001 was determined using Student's *t*-test.

To identify potential regulatory targets involved in periodontitis, we first analyzed the GSE10334 mRNA microarray dataset, consisting of 183 periodontitis and 64 healthy gingival tissue samples. Among the top 30 downregulated genes, PPI network analysis using the STRING database highlighted LOR as the gene with the most interaction nodes (Fig. 1A). To further validate the bioinformatic findings, we performed qRT-PCR analysis on clinical samples. The LOR mRNA expression was significantly lower in gingival tissues from patients having periodontitis compared to healthy controls (Fig. 1B). Consistent with these clinical observations, the LOR expression was also markedly suppressed in HPLF cells exposed to LPS, a well-established *in vitro* model of periodontitis injury (Fig. 1C). These findings suggest that LOR supports periodontal tissue homeostasis, and its downregulation in periodontitis may contribute to tissue breakdown during inflammation.

### LOR Overexpression Suppressed Inflammation and Promoted Cell Survival

**Fig. 2:**
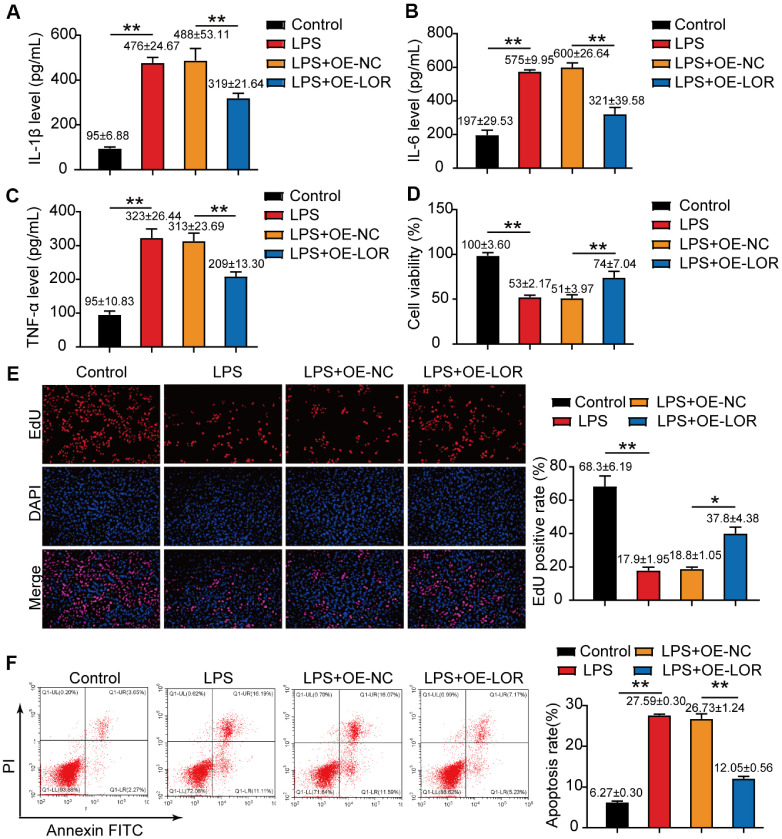
LOR overexpression mitigated inflammatory cytokine production, enhanced cell viability and proliferation, and suppressed apoptosis in LPS-stimulated HPLF cells. (**A–C**) ELISA assays were used to measure IL-1β, IL-6, and TNF-α levels in HPLF cells treated with LPS and/or transfected with LOR overexpression vector. (**D**) Cell viability was analyzed in aforementioned cells by using the CCK-8 assay. (**E**) EdU incorporation assays were used to assess cell proliferation in HPLF cells treated with LPS and/or transfected with LOR overexpression vector. (**F**) Apoptosis was evaluated via flow cytometry in these HPLF cells. All data (sample size = 3), obtained from three biological replicates, are presented as mean ± SD. *P < 0.05, **P < 0.001 was determined using one-way ANOVA.

To explore the functional significance of LOR downregulation in periodontal inflammation, we next examined the effect of LOR overexpression on our *in vitro* periodontitis model. The outcomes of ELISA revealed that LPS treatment dramatically elevated the amount of key pro-inflammatory cytokines (IL-1β, IL-6, and TNF-α) compared to untreated control cells (Fig. 2A-2C). Importantly, LOR overexpression in these cells notably reduced the levels of these cytokines, indicating that it exerts anti-inflammatory effects (Fig. 2A-2C). The CCK-8 assay showed that LPS treatment significantly reduced HPLF cells viability compared to untreated controls, while LOR overexpression substantially restored cell viability (Fig. 2D). The EdU incorporation assays demonstrated that LPS significantly inhibited HPLF cells proliferation compared to untreated controls. However, LOR overexpression markedly restored the proliferative capacity of these cells (Fig. 2E). Furthermore, LPS treatment induced a substantial increase in apoptotic cell populations, and LOR overexpression significantly suppressed this LPS-induced apoptosis (Fig. 2F). Collectively, these findings demonstrate that LOR not only limit inflammatory cytokine production but also preserve cell survival under inflammatory conditions, implicating it as important protective factor against periodontitis-associated tissue damage.

### METTL3 Overexpression Induced LOR Upregulation by Regulating Its m^6^A Modification

**Fig. 3:**
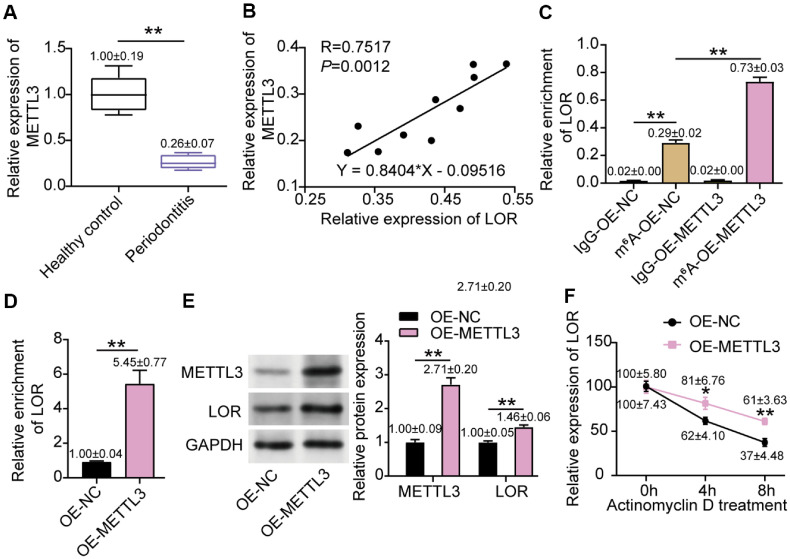
METTL3 enhanced LOR expression through m^6^A-mediated stabilization of its mRNA. (**A**) METTL3 mRNA levels were measured in clinical gingival tissues from periodontitis patients and healthy controls using qRT-PCR. **P < 0.001 was determined using Student's *t*-test. (**B**) Pearson correlation analysis was conducted to assess the association between METTL3 and LOR expression. (**C**) The relative m^6^A enrichment on LOR transcripts was determined by MeRIP assay following METTL3 overexpression, without IgG used for normalization. **P < 0.001 was determined using one-way ANOVA. (**D**) The LOR mRNA following METTL3 overexpression was analyzed by qRT-PCR. **P < 0.001 was determined using Student's *t*-test. (**E**) The LOR protein expression following METTL3 overexpression was analyzed by Western blotting. **P < 0.001 was determined using one-way ANOVA. (**F**) The mRNA stability assay was performed on actinomycin D-treated HPLF cells to determine the impact of METTL3 on LOR mRNA decay, with GAPDH used for normalization at each time. *P < 0.05, **P < 0.001 was determined using one-way ANOVA. All data (sample size = 3), obtained from three biological replicates, are presented as mean ± SD.

We then conducted *in vitro* experiments to investigate the interaction between METTL3 and LOR in periodontitis. The qRT-PCR analysis revealed that METTL3 mRNA expression was significantly lower in clinical samples as compared to healthy controls (Fig. 3A). The Pearson correlation analysis further revealed a strong positive relationship between METTL3 and LOR expression (Fig. 3B, R = 0.7517, P = 0.0012), supporting a direct regulatory association. To directly assess m^6^A modification on LOR transcripts, we performed MeRIP assays. The results elucidated that LOR mRNA was markedly enriched in the m^6^A fraction, and this enrichment was further enhanced by METTL3 overexpression (Fig. 3C). The qRT-PCR analysis illustrated that METTL3 overexpression led to a notable increase in LOR mRNA expression (Fig. 3D). Correspondingly, western blot outcomes revealed a marked increase in LOR protein levels, further confirming that METTL3 overexpression enhances LOR protein expression (Fig. 3E). The mRNA stability assay provided additional evidence of the functional significance of METTL3-mediated m^6^A modification. In the presence of actinomycin D, METTL3-overexpressing HPLF cells showed a significantly slower decay of LOR mRNA compared to controls, indicating enhanced mRNA stability (Fig. 3F). These results collectively establish that METTL3 promotes LOR expression by stabilizing its transcripts through m^6^A methylation, providing a novel mechanism for the regulation of periodontal cell responses.

### METTL3 Silencing Partially Attenuates the Protective Effect of LOR Overexpression in the LPS-Induced Periodontitis Cell Model

**Fig. 4:**
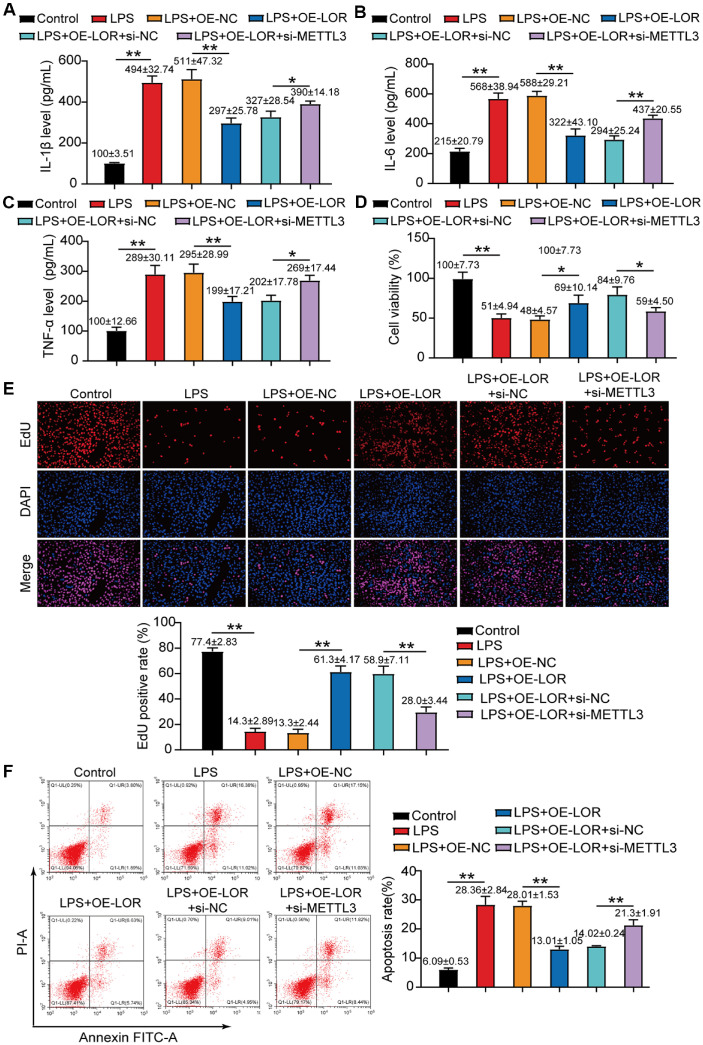
METTL3 silencing partially reversed the protective effects of LOR overexpression in LPS-stimulated HPLF cells. (**A–C**) ELISA assays were used to measure pro-inflammatory cytokine levels in LOR-overexpressing cells with or without METTL3 silencing under LPS treatment. (**D**) Cell viability in these cells was determined by CCK-8 assay. (**E**) Cell proliferation was assessed via EdU incorporation assay in LOR-overexpressing cells with or without METTL3 silencing under LPS treatment. (**F**) Flow cytometry was used to evaluate apoptosis under the same conditions. All data (sample size = 3), obtained from three biological replicates, are presented as mean ± SD. *P < 0.05, **P < 0.001 was determined using one-way ANOVA.

To confirm the biological relevance of the METTL3-LOR axis, we further evaluated the functional outcomes of silencing METTL3 in LOR overexpressed LPS-stimulated HPLF cells. The ELISA results revealed that silencing METTL3 in LOR-overexpressing cells partially reversed the cytokine reduction induced by LOR overexpression in the LPS-induced periodontitis model (Fig. 4A-4C). The CCK-8 assay outcomes indicated that silencing METTL3 in LOR-overexpressing cells partially negated the increase in cell viability observed with LOR overexpression in the LPS-induced periodontitis model (Fig. 4D). Moreover, EdU proliferation analysis showed that METTL3 silencing in LOR-overexpressing cells partially counteracted the enhanced cell proliferation typically seen with LOR overexpression in these cells (Fig. 4E). In parallel, flow cytometry data revealed that METTL3 silencing in LOR-overexpressing cells partially reversed the decrease in apoptosis induced by LOR overexpression (Fig. 4F). Altogether, these data highlight that METTL3 exerts its protective effects, at least in part, through the stabilization and functional restoration of LOR expression.

## Discussion

Periodontitis remains a prevalent and challenging chronic inflammatory disease that causes gradual degradation of the periodontal ligament, alveolar bone, and gingival tissues, ultimately leading to tooth loss and systemic complications [20, 21]. Deciphering the molecular mechanisms that drive this tissue destruction is imperative for developing targeted therapeutic strategies. Given the emerging importance of m^6^A modification in various diseases [9], we explored the role of m^6^A modification in LPS-induced periodontitis injury. Our study identified that LOR was downregulated in periodontitis, and LOR overexpression in LPS-treated HPLF cells reduced inflammatory cytokine production, enhanced cell viability and proliferation, and suppressed apoptosis. We also identified METTL3 as a key regulator of LOR expression, acting through m^6^A methylation to enhance its mRNA stability and promote gene expression at both transcriptional and translational levels. This novel protective pathway involving METTL3-mediated m^6^A modification of LOR provides new insights into both the basic molecular pathology and clinical management of periodontitis.

LOR is a key structural protein of the epidermis and plays an invaluable role in forming the cornified cell envelope, a structure critical for skin barrier function [22]. It is synthesized in the stratum granulosum and becomes cross-linked by transglutaminases during the terminal differentiation of keratinocytes [22]. It is encoded by the *LOR* gene, which is located within the epidermal differentiation complex (EDC) on chromosome 1q21, a region dense with genes involved in skin homeostasis and differentiation [23]. Mutations in *LOR* have been associated with inherited skin disorders such as Vohwinkel syndrome and progressive symmetric erythrokeratoderma, underscoring its essential role in epidermal biology [24]. A single previous study has shown that severe periodontitis was associated with disrupted LOR expression, but its specific role and mechanism in periodontitis did not reveal [25]. Our findings corroborate with above study and demonstrated that LOR was significantly downregulated in periodontitis tissues and in LPS-stimulated HPLF cells. The reduction of LOR correlated with increased inflammatory cytokine production, decreased cell proliferation, and enhanced apoptosis that are hallmarks of periodontal tissue injury [26]. Our data highlight that LOR downregulation may weaken tissue resistance to mechanical and microbial stresses, thereby amplifying periodontal breakdown.

METTL3, as a key m^6^A methyltransferase, along with METTL14 has been shown to influence the inflammatory response in periodontal ligament cells (PDLCs). It has been demonstrated that METTL3 expression was upregulated in LPS stimulated PDLCs and its knockdown resulted in suppressed expression of pro-inflammatory cytokines, indicating its role in modulating inflammatory signaling pathways [27]. In contrast, our findings highlight a significant downregulation of METTL3 in LPS-stimulated HPLF cells. This apparent contradiction may stem from cell-type-specific differences between PDLCs and HPLFs. While both are derived from the periodontal ligament, HPLFs are a more defined fibroblast population, whereas PDLCs represent a heterogeneous mix of fibroblasts, epithelial cells, stem cells, and immune cell subsets, which may contribute to a more inflammatory milieu under stimulation. Moreover, the local inflammatory context and the nature of stimuli may influence METTL3's function. Differences in LPS source, concentration, and exposure duration can yield varying outcomes on METTL3 expression and activity. Additionally, the downstream effects of METTL3 are highly dependent on the availability and specificity of m^6^A readers and erasers within a given cell type. For instance, divergent expression patterns of m^6^A readers such as YTHDF1/2 or IGF2BP2 may dictate whether METTL3 exerts pro- or anti-inflammatory effects in periodontal cells. Interestingly, our findings are also partially supported by studies in other periodontal cell types. For example, a study reported downregulation of METTL3 in LPS-treated human periodontal ligament stem cells (hPDLSCs), where its overexpression promoted osteogenic differentiation via the miR-141-3p – ZEB1 axis [17]. While that study focused on osteogenesis rather than inflammation, it underscores the functional diversity of METTL3 depending on cellular identity and biological context. In the realm of bone regeneration, METTL3 is known to stabilize transcripts such as Runx2 in hPDLSCs and modulate osteogenic lncRNAs like lncRNA CUTALP in periodontal mesenchymal stem cells via m^6^A modification [16, 28]. Taken together, these observations suggest that METTL3 may play dual and context-dependent roles in periodontal health—promoting inflammation in PDLCs while exhibiting protective or regenerative functions in HPLFs. Our study provides novel insight into the anti-inflammatory potential of METTL3 in HPLFs, emphasizing the need for further investigation into the cell-specific regulatory networks that govern m^6^A dynamics in periodontitis.

Mechanistically, we revealed that METTL3 enhanced LOR expression by stabilizing its mRNA transcripts through m^6^A methylation. Importantly, our study is the first to identify METTL3-mediated m^6^A methylation as a regulatory mechanism for LOR in the context of periodontal inflammation. We have established m^6^A-modified LOR as a novel regulator of inflammation and tissue survival. Restoration of LOR expression not only suppressed LPS-induced production of TNF-α, IL-6, and IL-1β but also promoted HPLF cells proliferation and prevented apoptosis. This dual effect suggests that LOR acts as a central mediator coupling structural tissue integrity with inflammatory control. Clinically, our findings propose that enhancing the METTL3-LOR axis might offer a promising therapeutic approach for periodontitis. Current periodontal therapies largely focus on mechanical debridement and antimicrobial interventions [29], which do not address underlying molecular defects. Targeted modulation of m^6^A regulators, or direct supplementation of LOR expression, could preserve periodontal fibroblast function, limit inflammatory damage, and promote tissue regeneration. Furthermore, LOR or METTL3 levels could serve as diagnostic biomarkers for early detection of periodontal tissue susceptibility before clinical manifestations become severe.

We certainly acknowledge the limitations in the present study. While our *in vitro* experiments using HPLF cells provided valuable insights into the METTL3–LOR axis in regulating fibroblast inflammatory responses, they do not fully replicate the complexity of the periodontal microenvironment, which includes immune cell infiltration, vascular changes, and dynamic tissue remodeling. These factors play critical roles in the pathogenesis of periodontitis and may influence the behavior of METTL3 and LOR *in vivo*. Therefore, further *in vivo* studies, LPS-induced periodontitis model in mice, are necessary to validate the protective role of METTL3 and LOR under physiologically relevant conditions. Additionally, identifying upstream regulators of METTL3 activity and elucidating potential interactions with other m^6^A 'reader' or 'eraser' proteins would provide a more comprehensive understanding of RNA modification dynamics during periodontal inflammation.

In conclusion, we identified that METTL3-mediated m^6^A modification stabilizes LOR mRNA, which in turn suppresses inflammatory cytokine production, preserves fibroblast proliferation, and inhibits apoptosis in the context of periodontitis. These findings reveal a previously unrecognized protective pathway and imply that targeting m^6^A RNA methylation or restoring LOR expression warrants further investigation as a potential approach for preventing or managing periodontitis

## Supplemental Materials

Supplementary data for this paper are available on-line only at http://jmb.or.kr.
