# Supplementary material for: LOR Regulated by METTL3 Alleviates Lipopolysaccharides-Induced Periodontitis Injury
Source: J Microbiol Biotechnol. 2025 Aug 18;35:e2505016. doi: 10.4014/jmb.2505.05016 (PMC12375539; doi:10.4014/jmb.2505.05016)
Supplement: Supplementary file 1 [file jmb-35-e2505016-supple.pdf]

J. Microbiol. Biotechnol. 2025. 35: e2505016
https://doi.org/10.4014/jmb.2505.05016

Supplementary Tables

Table S1. Clinical characteristics.

Characteristics	Healthy control （n=10）	Periodontitis （n=10）	*P* value
Age(yeas)	46.45±13.12	47.76±11.49	0.315
Sex（Male/Female）	5/5	6/4	0.332
Dietary favor (light/heavy)	6/4	3/7	0.301
GI	0.62±0.13	3.02±0.64	＜0.01
PI	0.92±0.43	2.72±0.85	＜0.01
PD (mm)	1.67±0.55	5.04±1.14	＜0.01
CAL (mm)	0.58±0.19	4.12±1.02	＜0.01

Abbreviations：GI (gingival index)；PI (plaque index)；PD (probing pocket depth)；CAL (clinical attachment level).

Table S2. Real-time PCR primer sequences.

Gene name	Sequence
LOR	Forward 5'-CTCTGTCTGCGGCTACTCTG-3'
	Reverse 5'-CACGAGGTCTGAGTGACCTG-3'
METTL3	Forward 5'-AAGCTGCACTTCAGACGAA-3'
	Reverse 5'-GGAATCACCTCCGACACTC-3'
GAPDH	Forward 5'-GCACCGTCAAGGCTGAGAAC-3'
	Reverse 5'-TGGTGAAGACGCCAGTGGA-3'
